# IoT device fabrication using roll-to-roll printing process

**DOI:** 10.1038/s41598-021-99436-0

**Published:** 2021-10-07

**Authors:** Thanh Huy Phung, Anton Nailevich Gafurov, Inyoung Kim, Sung Yong Kim, Kyoung Min Kim, Taik-Min Lee

**Affiliations:** 1 Department of Printed Electronics, Korea Institute of Machinery and Materials (KIMM), 156 Gajeongbuk-Ro, Yuseong-Gu, Daejeon, 34103 Republic of Korea; 2 Department of Nanomechatronics, Korea University of Science and Technology (UST), 217 Gajeong-ro, Yuseong-gu, Daejeon, 34113 Republic of Korea; 3 Department of Electronics Engineering, Korea Polytechnic University, 237 Sangidaehak-ro, Siheung-Si, Gyeonggi-Do, 15073 Republic of Korea; 4 Department of Advanced Materials Engineering, Korea Polytechnic University, 237 Sangidaehak-ro, Siheung-Si, Gyeonggi-Do, 15073 Republic of Korea

**Keywords:** Engineering, Techniques and instrumentation

## Abstract

With the development of technology, wireless and IoT devices are increasingly used from daily life to industry, placing demands on rapid and efficient manufacturing processes. This study demonstrates the fabrication of an IoT device using a roll-to-roll printing process, which could shorten the device fabrication time and reduce the cost of mass production. Here, the fabricated IoT device is designed to acquire data through the sensor, process the data, and communicate with end-user devices via Bluetooth communication. For fabrication, a four-layer circuit platform consisting of two conductive layers, an insulating layer including through holes, and a solder resist layer is directly printed using a roll-to-roll screen printing method. After the printing of the circuit platform, an additional layer of solder paste is printed to assemble the electrical components into the device, inspiring the fully roll-to-roll process for device fabrication. Successful IoT device deployment opens the chance to broaden the roll-to-roll fabrication process to other flexible and multilayer electronic applications.

## Introduction

The blooming of Industry 4.0 has opened the wide use of IoT (Internet of things) in various areas, from daily life to science, technology, and manufacturing^1–3^, requiring the mass production of IoT devices to meet market demands. For IoT devices, flexible printed electronics is a good option for device fabrication due to its various advantages such as being thin, lightweight, and cost-efficient^4–6^. The cost-effectiveness and rapid production time of flexible electronics can quickly adapt to rapidly changing technology and consumer requirements, while its flexibility and lightness facilitate working in a variety of conditions and workspaces. Moreover, the use of flexible substrates creates the possibility to fabricate the devices using roll-to-roll (R2R) processes, which could reduce significantly the production time and cost for mass production.

The basic components of an IoT device consist of the power supply, the sensing part with sensors, the controller and data processing part, and the wireless communication to cloud services or other end-user devices such as mobile phones and computers^7^. So far, researchers and scientists have successfully developed the printed sensors for flexible electronics^4,8–13^. The sensors are then integrated into the printed circuit platforms with other electronics components such as filters, amplifiers, and microcontrollers to form the complete devices^14^. With the grown of printed electronics technology, the use of printed devices capable of wireless communication has been increasing for applications that require compact size and remote distance sensing or actuation, such as wearable, healthcare devices^15^, or robotics^16^. For this purpose, several approaches have been proposed. For example, Song et al.^17^, Gao et al.^18^ created a wearable sweet sensor and integrated it with a conventional FPCB (flexible printed circuit board) capable of BLE (Bluetooth Low Energy) transmission. Instead of using FPCB as a circuit platform, Wang et al.^19^ created a wireless temperature sensing device with a temperature sensor and circuit platform both by screen printing. Recently, Bhattacharjee et al.^20^ created a chipless antenna including a temperature sensor, which opens changes to create devices without external integrated communication modules.

In this study, our approach is to print multilayer circuit boards as circuit platforms for the devices using R2R printing, then assemble commercially available solid-state or flexible printed devices to implement the IoT devices, which has been well-known as “hybrid fabrication”^4,5^. By this approach, the number and types of sensing or electronics components become flexible. Note that although the sensor could be printed integratedly using printing technologies, practical devices may require different types of sensors. Accordingly, different types of materials and processes are required, which may hinder the efficiency of the whole fabrication process. For device making, we implemented a R2R fabrication of hybrid, multilayer printed circuit boards (PCBs) that could be used as circuit platforms for IoT applications or other electronics applications in general, where the designs for the conventional rigid circuit could be transferred directly to use with the R2R printing process. Note that the mounting of electronics could be automatic, which inspires a fully R2R process for the fabrication of the devices.

For the printing of circuit platforms, there have been efforts to print the multilayer circuits on flexible substrates so far. As simple trials, the circuits were specially designed with few intersection points between the layers, then the dielectric materials were printed on small areas for insulating^6,21–23^, which could avoid the use of via-holes. However, the needs for special design on circuits make the method less comparative to the conventional PCB making methods. The circuits designed for conventional methods could not be interchanged with the printing methods, and due to the simplicity of the circuit, fewer functions could be used. To overcome the limitations, efforts have been made to implement the multilayer circuit board with via holes. For instance, Park et al.^24^ developed a potential process for complex circuit printing using R2R gravure printing. After printing the circuit, the via holes were drilled by laser spot, then filled using jet printing. The required steps of the process increase and appear outside the R2R process. Moreover, in the case of PCB with more than 2 layers, several circuits were printed separately, then drilled and stitched together^25^. In another approach for stretchable PCB, the printed layer was fully spin-coated by the photosensitive insulator, then the via holes were made by photolithography on coated layer^26^. In this case, the cost of the product increases due to the requirement of materials, and the coating and printing are 2 sole processes. Recently, Phung et al.^27^ implemented a hybrid fabrication method for four conductive layers PCB, where the core circuits were double-sided printed on flexible substrate using R2R screen printing undergoing stacking of other layers onto the core circuits. Here, the drilling process was still necessary to create the via-hole, and electroplating was used to create through-hole connections. In general, the limitation of those approaches is the creation of via-holes and the interconnection between the layers. No matter how the via-holes are drilled, the creation and interconnection of the holes are time-consuming, which is proportional to the number of the holes. Moreover, the through-hole connectivity requires another process such as filling or electroplating, which requires more time and effort.

In this work, we printed the multi-layer circuit platform by using R2R screen printing, in which the via-holes and interconnection were printed directly through the overlay layers. To show the capability of broadening electronic applications, the circuits were converted from conventional rigid PCB design into a form for R2R printing. Here, the challenge of R2R printing for multilayer printing is the alignment of the overlay printed layers (i.e. registration control) causing when the flexible polymeric substrate faces tension and thermal heating during sinter or annealing processes. To overcome the issues, we used an institutional-developed R2R system that included a vision-based alignment method for real-time printing, along with the passive compensation method to modify the designed circuit^27–29^. Moreover, the ink and printing factors were investigated to match the printing performance and alignment. Here, the circuit with four aligned layers was successfully printed for IoT application. After printing the circuit platform, the solder paste was screen-printed to mount the electronics components, sensors, and BLE module to form the completed IoT devices.

## Results and discussion

### Overall fabrication process

**Figure 1:**
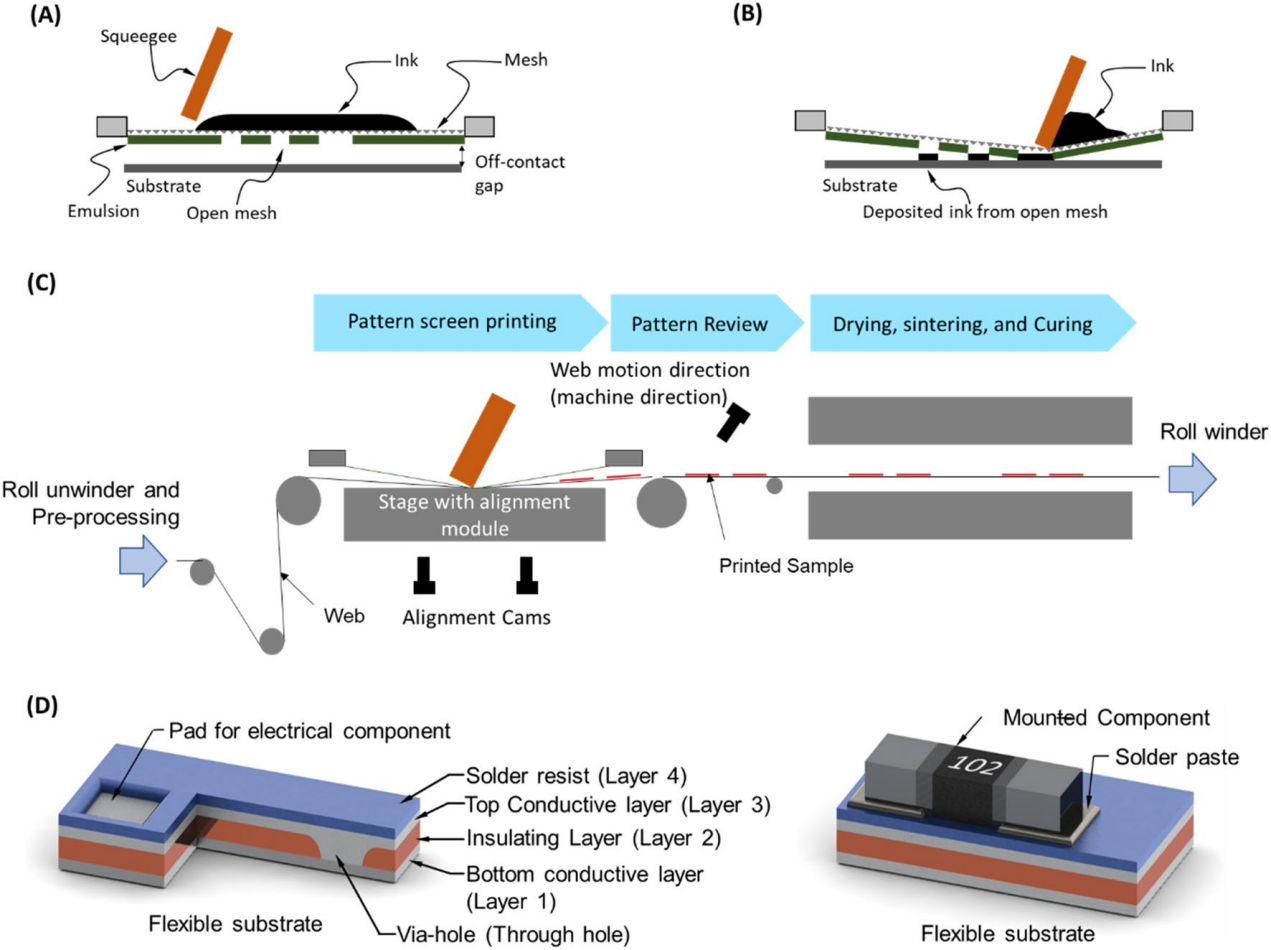
Printed circuit board fabrication. (**A**, **B**) principle of screen printing, (**C**) roll-to-roll screen printing process, (**D**) Basic structure of the printed circuit board and the mounted electronic components with printed solder paste.

The overall fabrication process for the IoT devices in this study is illustrated in Fig. 1. The printing method used throughout the process is screen printing. Screen printing is a popular printing method having a simple principle as described in Fig. 1A, B. ^30^. The basic component of the printing method is the screen mesh, which is covered by an emulsion layer. In order to print a designed pattern (circuit), the pattern is converted into the open parts on the emulsion layer. For printing, the mesh is placed above the substrate at a gap distance (off-contact), and ink is scrapped on the mesh. Sequentially, a squeegee applies a printing pressure on the mesh and travels, causing the ink to be deposited on the substrate through the open mesh.

For scalable and mass production, screen printing was used as a R2R process as shown in Fig. 1C, where the flexible substrate was prepared as a roll of polymeric film (web) to feed and print continuously. In this way, the printing could be aligned and monitored automatically during printing^27,29^. This approach could gain another advantage, which is the post-printing processes such as drying, sintering, curing, or crystallization for the functional materials could be performed inline thus reduce significantly the processing time and experimental setup (“Experimental” section).

By using R2R screen printing with inline sintering and curing system, PCBs with the structure depicted in Fig. 1D were fabricated. Printed PCBs included four printed layers of which two are conductive. First, a conductive layer (bottom layer) was printed on the substrate, followed by the insulating layer. Here, the via-holes could be printed directly on the substrate by designing the insulating layer, which avoids complicated and time-consuming processes such as drilling and electroplating. The top conductive layer was printed on the insulating layer and interconnected with the bottom layer by filling the via-holes as shown in Fig. 1D. Finally, a solder resist (solder mask) layer was printed to facilitate the soldering process and protect the circuit from external harsh conditions. The solder-resist layer consists of openings that are used as the pads to mount electrical components (Fig. 1D). The mounting of electronic components is compatible with the R2R process by using screen printing to print the solder paste, undergoes with automatically mounting component inline. By combining the processes, a fully R2R process for the fabrication of IoT and other electrical devices, in general, could be archived.

**Figure 2:**
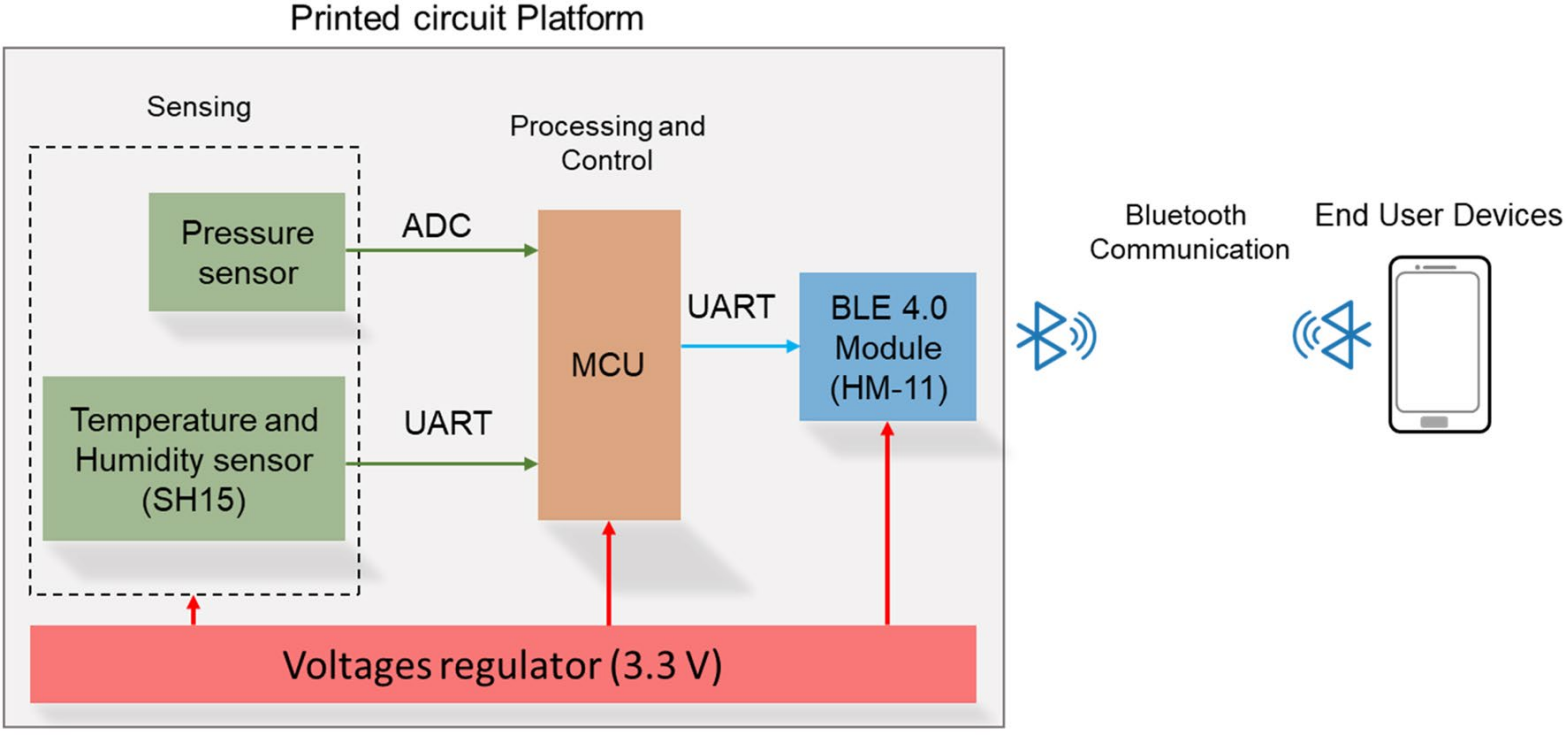
IoT device structure and components.

Figure 2 shows the basic components of the IoT device fabricated in this study. The device included a pressure sensor and a temperature and humidity sensing module. The sensing signal is processed by a microcontroller before being sent to the BLE module for wireless transmission. All the components were mounted on a 4-layers printed circuit platform. In order to print the circuit platform, the Gerber files (design file for fabrication) were converted into the mask form, then scaled with the alignment factors for registration control. (Experimental Section).

### Printing of conductive and insulation materials

Consider that the printed circuit should withstand high temperatures during soldering to assemble the electronic components, which might include the impacts of soldering tip, epoxy-based silver paste ink purchased from FP (FP Co. Ltd., Busan, Rep. of Korea) was used for the printing of the conductive layers. The use of epoxy-based ink has another advantage of improving the adhesion between the conductive layers with the substrate as well as the insulating layer. Note that in circuit printing using screen printing, extra adhesive layers may be used^31^ to enhance the adhesion between the printed layers and the substrate. However, the polymeric substrate in the R2R process is subject to the tension and thermal effects^29^. As a result, the extra printed layers could cause alignment and printing more difficult, which is not recommended.

**Figure 3:**
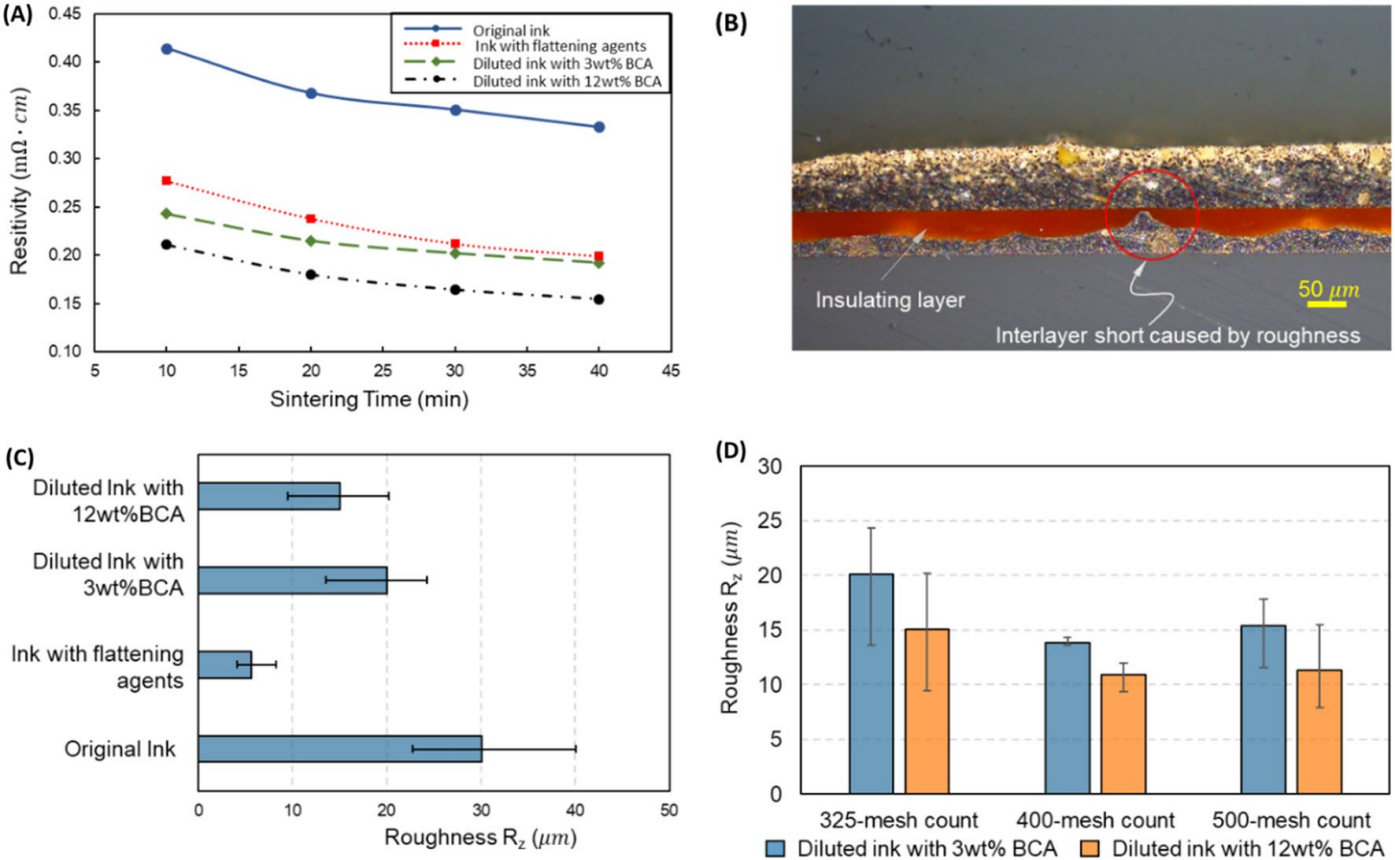
Conductive layers patterning. (**A**) the resistivity of printed patterns using different inks, (**B**) roughness causing interlayer shortage, (**C**) roughness of the printed patterns with respect to the inks, and (**D**) roughness regarding the screen mesh.

The printing using epoxy-based ink faced 2 issues: roughness and conductivity. Figure 3 shows the roughness and conductivity of the printed patterns after sintering. Initially, the screen meshes with 325-mesh count (i.e., meshes with 325 threads per inch square) were used for printing. Note that for R2R printing, high temperature and longtime sintering could result in deformation of the web (substrate), or improper registration control. Therefore, the sintering temperature and duration should be limited. In Fig. 3A, the printed patterns were sintered at 130 °C. Another important factor is the roughness. The high roughness should be avoided because it could cause interlayer shortage without notices as shown in Fig. 3B. To solve those issues, 3 methods have been investigated, including thinning the ink by diluting with BCA (*Butyl Cellosolve Acetate*), adding the flattening agent, and controlling the mesh and printing conditions.

Figure 3A shows that the conductivity of the ink improved significantly after ink modification. In the case of original epoxy ink, the surface roughness was high, leading to the nonuniformity of the pattern thickness (Fig. 3C). As a result, the measured resistance and overall resistivity of the printed pattern could be higher. In the case of adding the flattening agent and BCA, the thickness of the printed layer became more uniform. Consequently, it could increase the conductivity of the pattern. However, the added flattening agent could remain if the sintering condition is not proper while BCA could evaporate easily during sintering.

**Figure 4:**
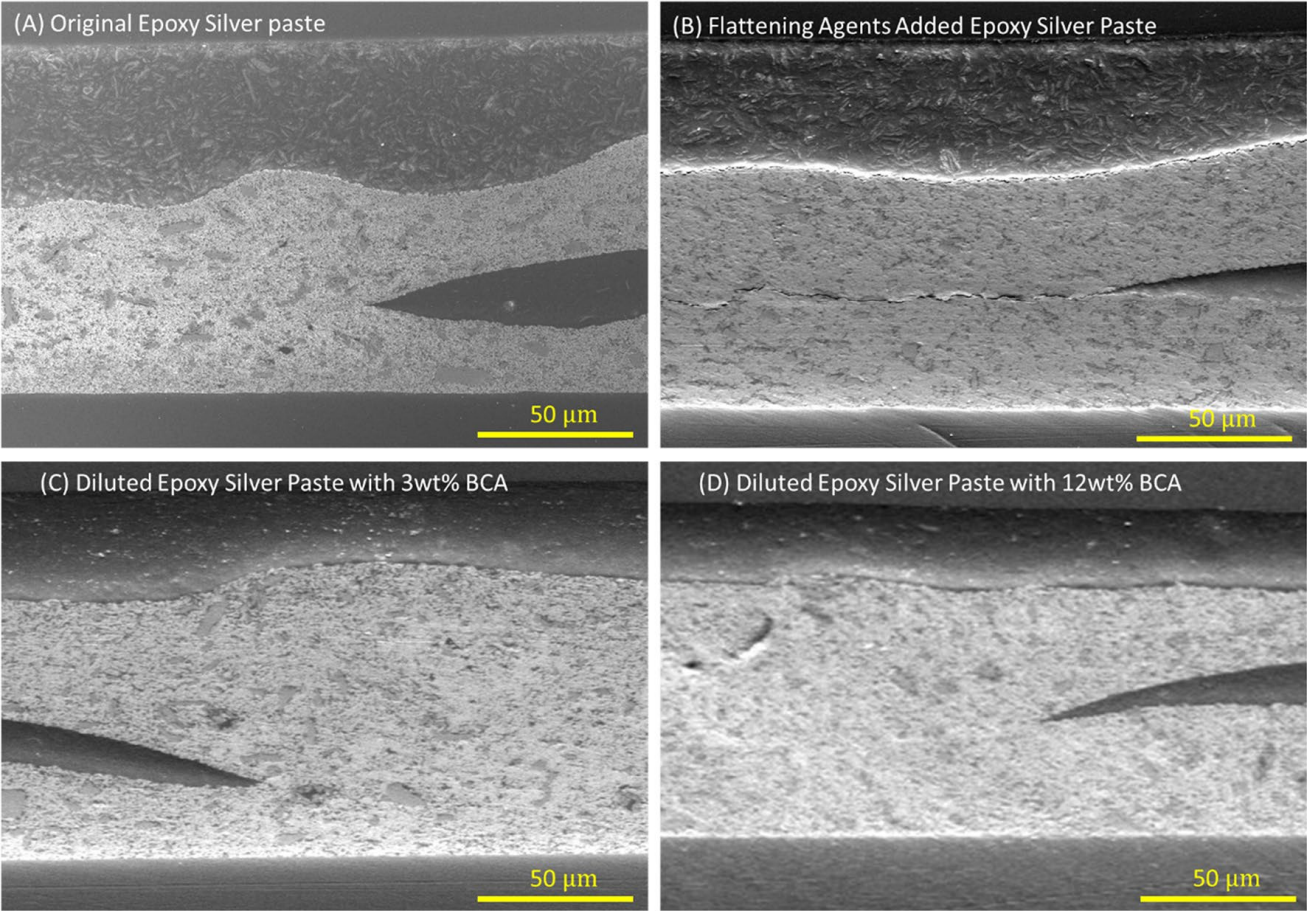
Coss-section between the top and bottom conductive layers at the via-holes. (**A**) Original Epoxy Silver Paste, (**B**) Epoxy Silver Paste with added flattening agent (**C**) Diluted Epoxy Silver Paste with 3wt% BCA, and (**D**) Diluted Epoxy Silver Paste with 12 wt% BCA.

Figure 3C shows the roughness of the printed pattern with respect to the inks. As shown in Fig. 3C, the ink added flattening agent had the best results of about $R_{z} \approx 6 \mu m$ (averages height from the peaks to the valleys of the surface profile). In the case of using the diluted solvent (BCA), the viscosity of the ink reduced, resulting in ink leveling. Accordingly, the ink diluted with more solvent may produce better printing roughness. However, when the flattening agent was added, the cracks occurred at the contact position between the top and bottom layers inside the via-holes as shown in Fig. 4. This is not allowable for the working of the final product (Fig. 4). In contrast, the interlayer connections could be printed without any defects by original and diluted inks as shown in Fig. 4. Since the use of the flattening agent may not be suitable for overlay printing, only diluted inks with BCA should be used to reduce the roughness of the printed patterns.

To improve the roughness of the conductive layer without using the flattening agent, finer meshes were investigated for printing. Figure 3D shows the influence of the mesh count on the roughness of the printed layer. The finer mesh count has a shorter mesh pitch and thinner thread. As a result, the printed surface became smoother as shown in Fig. 3D. However, the extra-fine mesh count (500), too short mesh pitch may hinder the ink transfer through the mesh, and the roughness of the printed patterns worsen. From the experiments, the diluted ink and screen mesh of 400-mesh count were selected for the printing of the bottom layer. Note that for the top layer, the roughness may not cause significant issues, therefore, a lower mesh count is usable.

**Figure 5:**
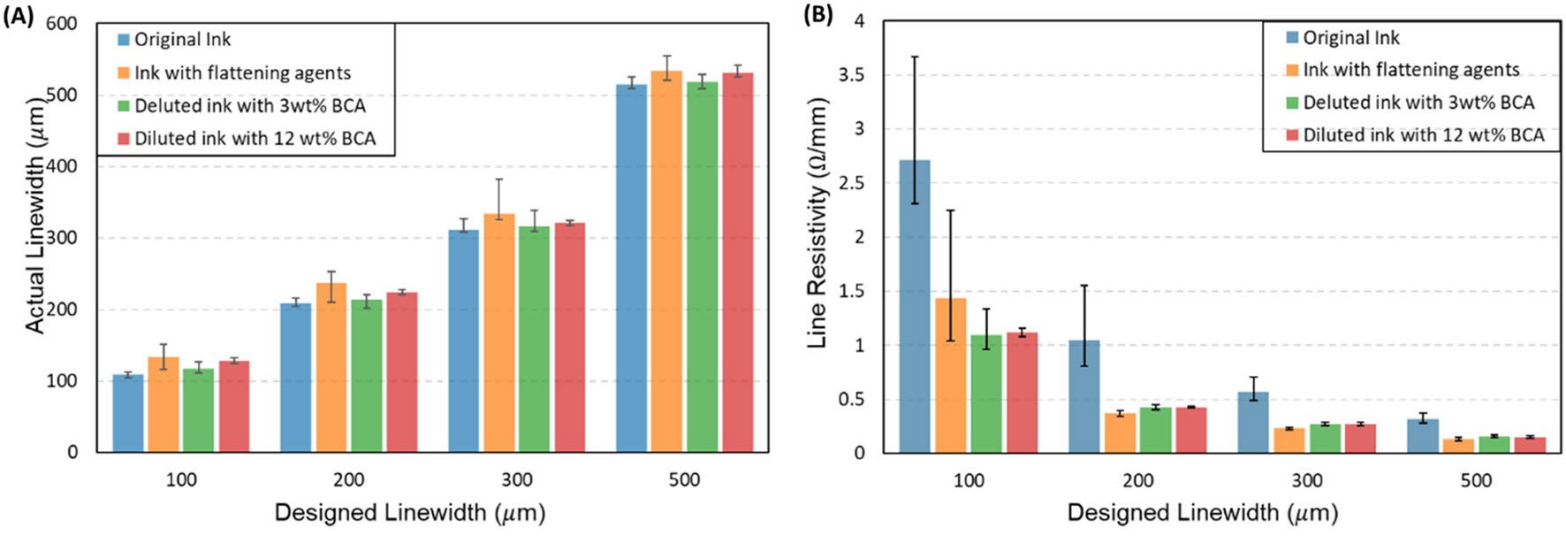
Line width and line resistivity of the printed line using conductive ink.

For practical printing and circuit design, the resistance and the limit feature size of the conductive patterns should be considered. Figure 5 shows the line resistivity and actual line width of the printed line according to the design with the line thickness of about 20 μm. Even though screen printing could print the pattern with a width smaller than 100 μm, the designed linewidth should be large enough for conductivity and reliability. As shown in Fig. 5, the line width should be larger than 100 μm. For this notice, the linewidth from 200 μm and the line interval from 500 μm are recommended. Consequently, the design for SMD (surface mounting devices) with feature size from 1005 and 1608 (metric, i.e. 5 mm × 10 mm and 16 mm × 8 mm, or imperial 0402 (4 mils × 2 mils) and 1005 (10 mils × 5 mils)), and most QFP ICs (quad flat package integrated chips) could be used.

**Figure 6:**
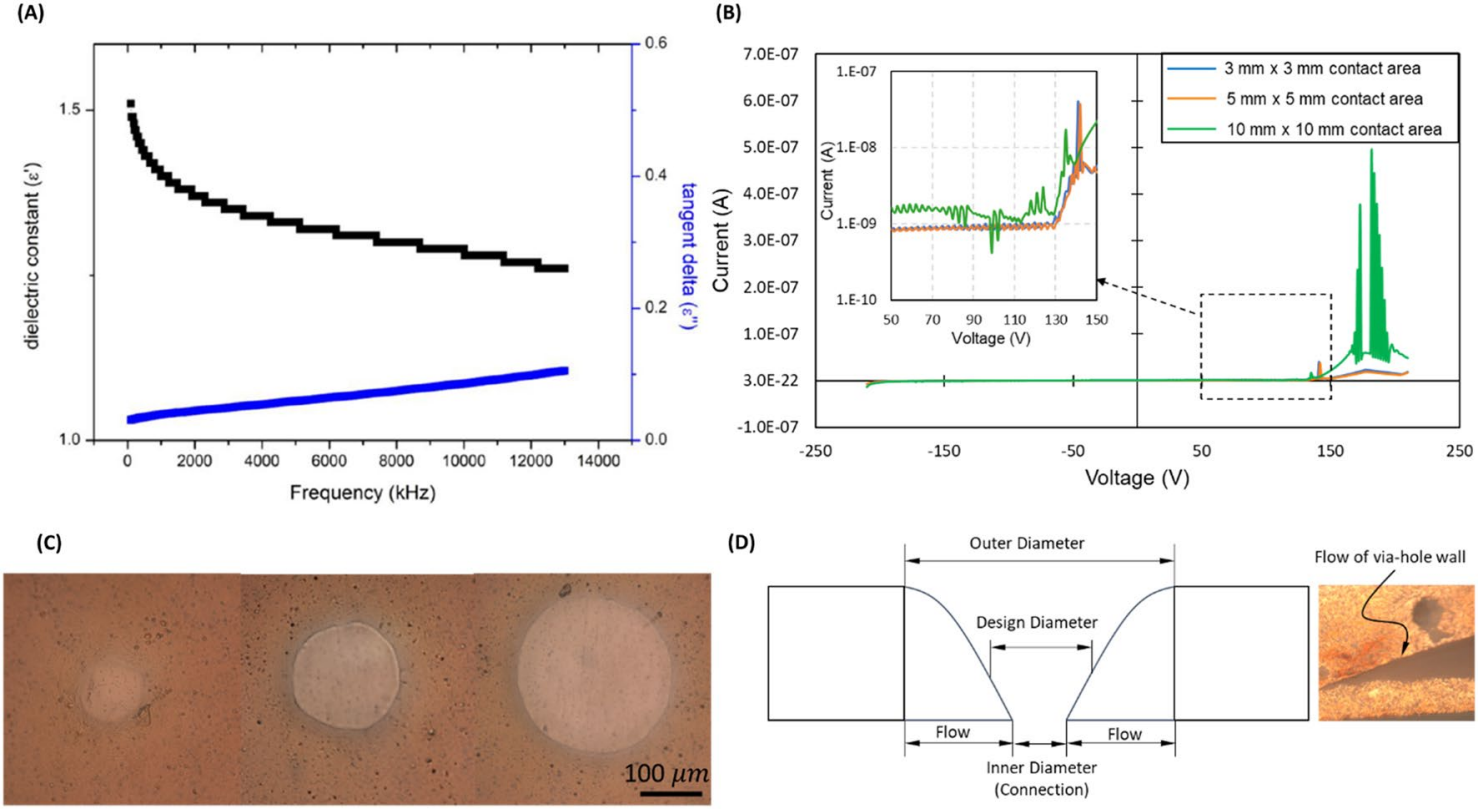
Insulating ink properties and via-hole printing. (**A**) Dielectric properties of the insulating ink, (**B**) IV-curve and breakdown voltage of the printed circuit, (**C**) Printed via-holes with diameters of 100, 200, and 300 μm respectively, and (**D**) printing of via-hole.

In the case of fully printed multilayer circuit boards, the printing of the insulating layers and via-holes is important. In this study, a thermal plastic insulating ink (SRF-300PIY, Seoul Chemical Research Lab Co., Ltd, Rep. of Korea) was used. Figure 6 shows the ink properties and printing results of the insulating layer. According to Fig. 6B, the printed layer (having a thickness of 25 μm on the printed conductive layer with a roughness of ~ 15 μm) was well insulated until broken down at about 130 V, which is sufficient for IoT devices. For via-hole printing, the holes with diameters down to 100 μm could be printed as shown in Fig. 6C. However, the design for the via-holes should be large enough for interconnection between conductive layers. After printing, the walls of the via-holes are not perpendicular to the substrate surface as in the case of drilling. Due to the flow of printed ink, the walls tend toward the center of the holes, which makes the inner holes for interconnection become smaller and the outer diameters become large than the original design as shown in Fig. 6D. This flow could be severe if the ink viscosity is low, or the printed thickness is high. As a result, if the design diameter is too small, the holes could be covered and the openings for interconnection may not be sufficiently conductive. For the printing conditions in this study, the design diameter is suggested to be more than 300 μm in order to ensure connectivity. Note that the topper conductive layer should be designed to cover the outer diameter of the printed holes to fill the via-holes (Fig. 6D).

### IoT device implementation

**Figure 7:**
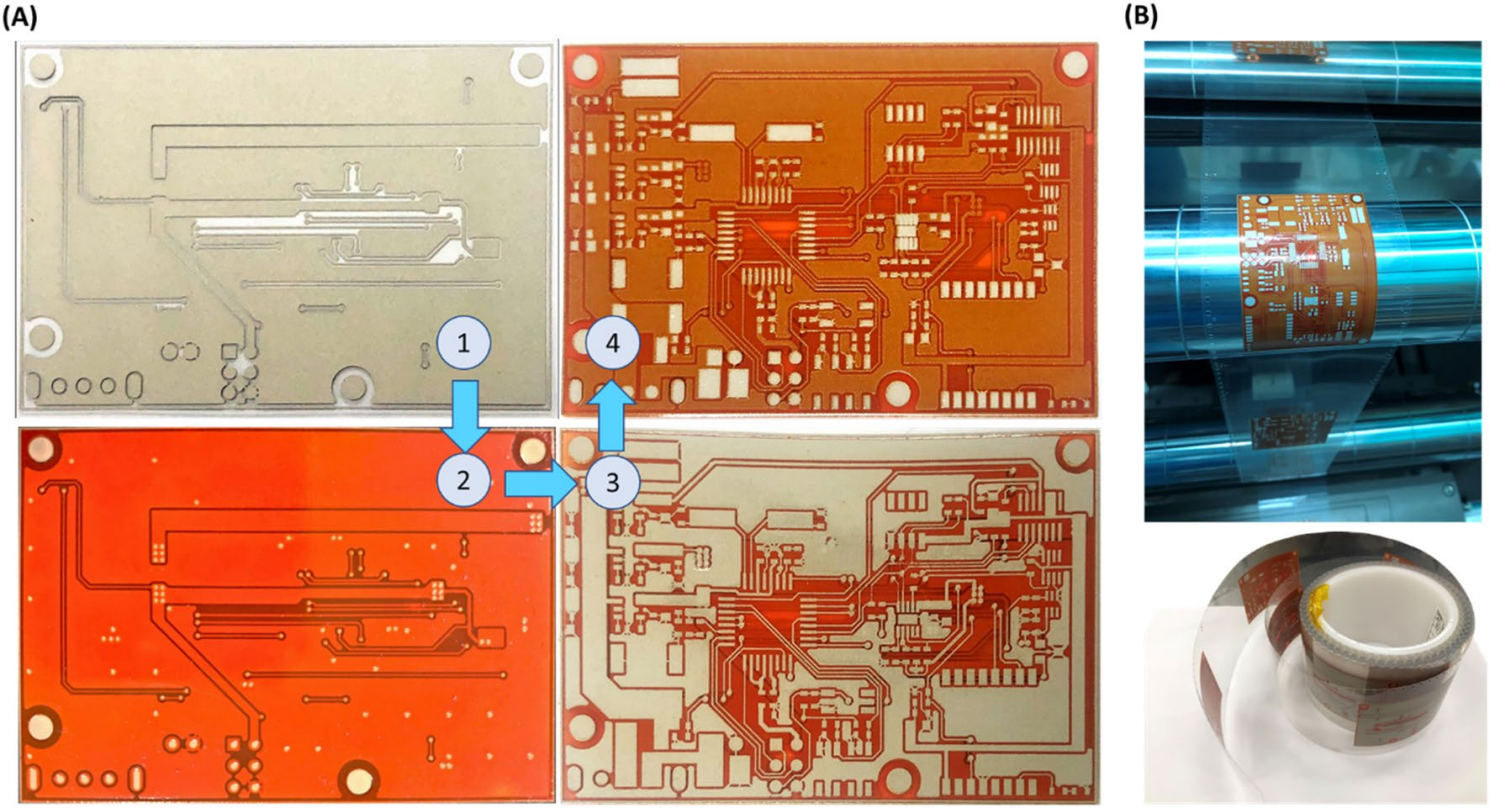
IoT circuit platform printing using roll-to-roll screen printing. (**A**) 4-layer printing of IoT circuit platform, and (**B**) the printed product on the web and final printed circuit roll.

**Figure 8:**
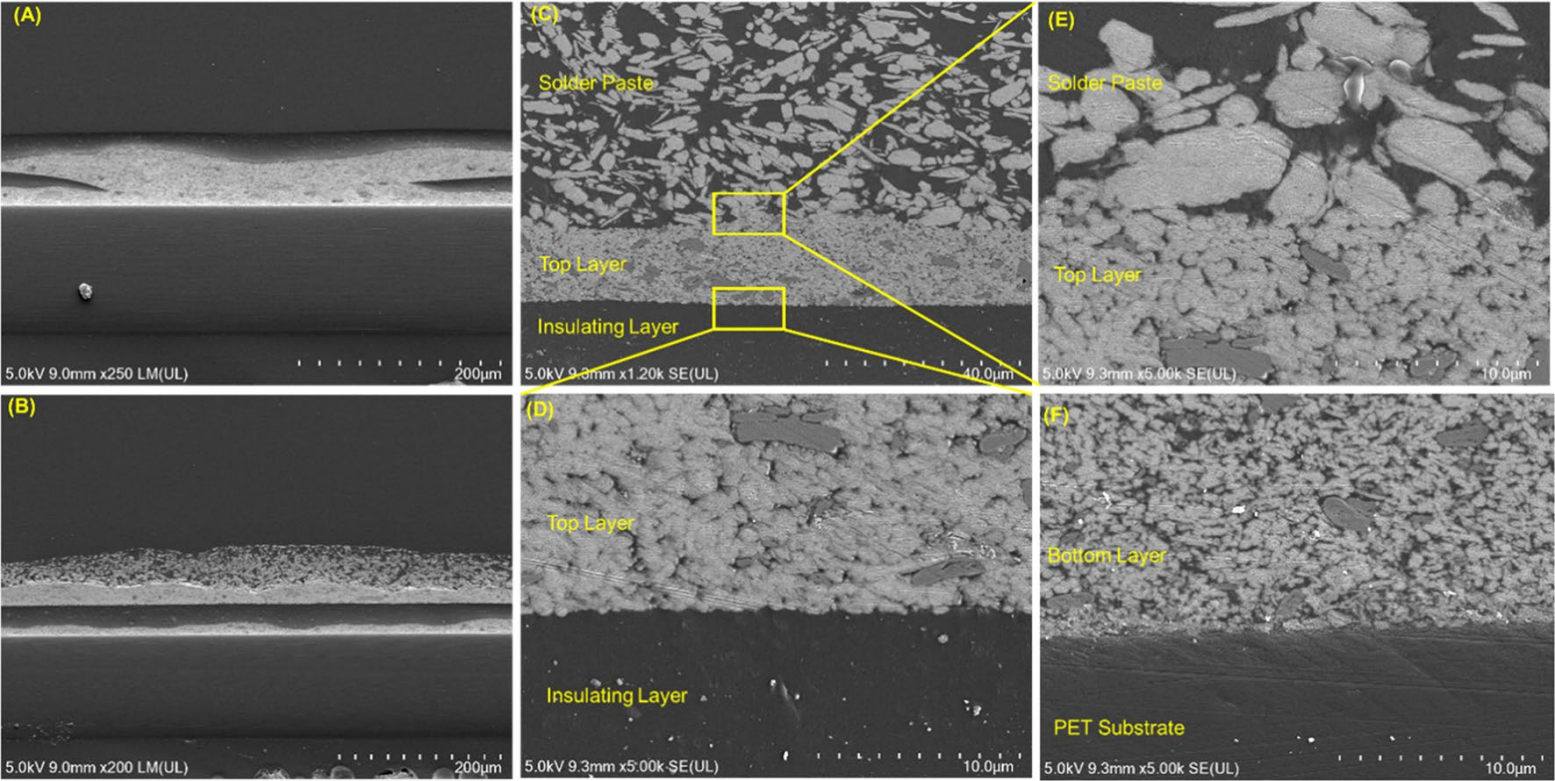
Microstructure of the printed layers. (**A**) Cross-section of the printed 4-layer circuit at the via-hole, (**B**) Cross-section at the pad to mount electronic components with printed solder paste, (**C**–**E**) connection between solder paste and the top conductive layer, the top conductive layer and the insulating layer, and (**F**) Connection of the bottom conductive layer and PET substrate.

**Figure 9:**
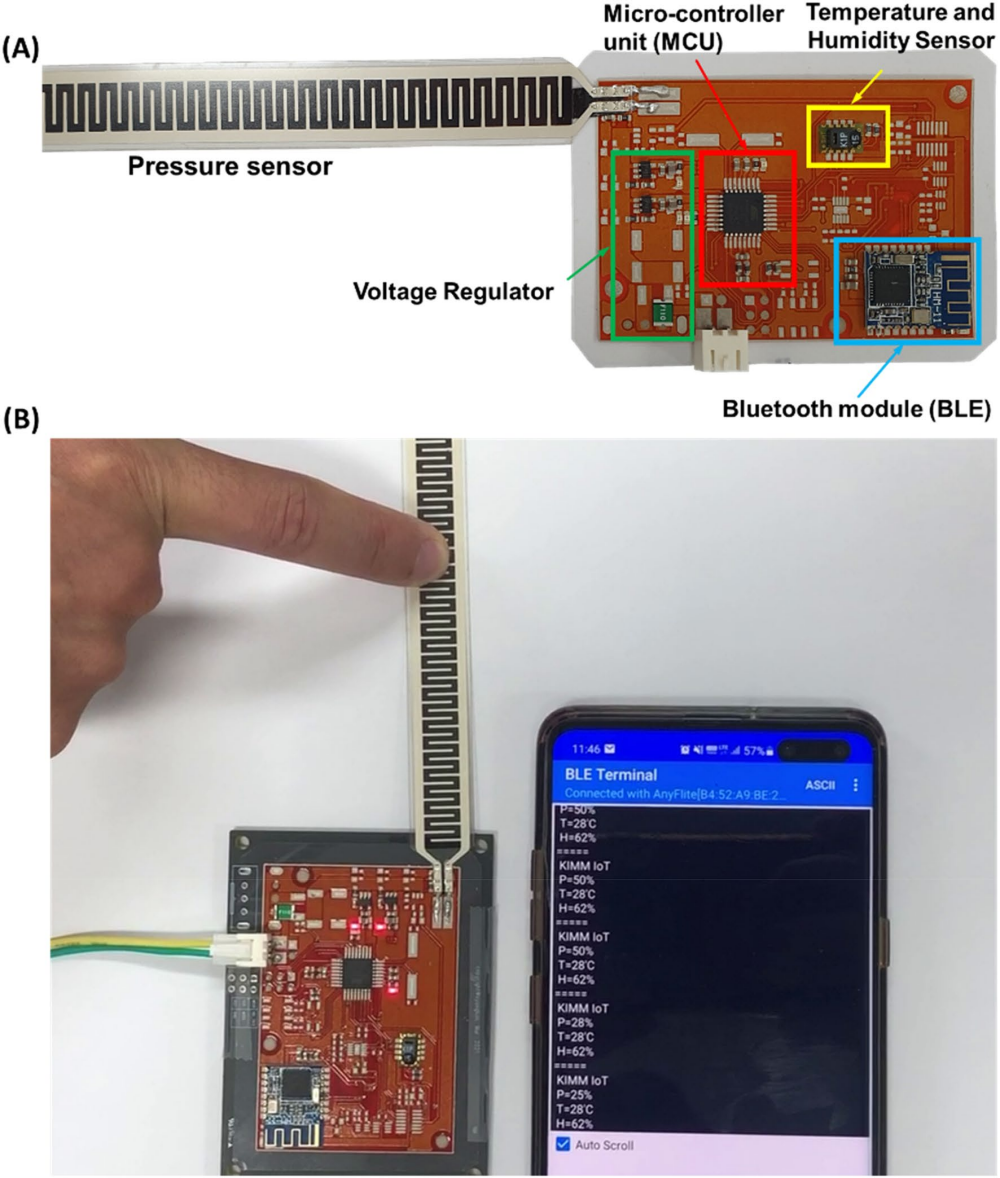
Implemented IoT devices. (**A**) The structure of the implemented IoT devices, and (**B**) Testing the implemented IoT devices.

After investigating the materials and printing performance, the circuit platform for IoT devices was printed as shown in Fig. 7. In our approach, the circuit platform was printed using R2R screen printing by overlapping 4 printing layers as shown in Fig. 7A. Here, the registration control process has been performed to align the printed layers^27,29^ (Experimental section). Figure 7B show the printed roll for the next stages. After printing the circuit platform, the solder paste was screen printed and the electronic components were mounted. Figure 8 shows the microstructure of the printed platform. As shown in Fig. 8, the connection between the printed layers as well as between the bottom layer and the substrate were well adhesive. Moreover, the layers including the solder paste layer had no cracks nor deformation and were suitable for practical use. The final IoT device was successfully implemented as shown in Fig. 9. As shown in Fig. 9A, the implemented IoT device has a temperature and humidity module (SH-15) and a flexible resistive pressure sensor. A microcontroller unit (Atmega8a) controls the devices and processes the data, then sends the information to a Bluetooth module (HM-11) to communicate with other devices as shown in Fig. 9B.

## Conclusion

In summary, we fabricated the IoT devices with the printed circuit platform including four layers using R2R screen printing. For printing of the conductive layers, the epoxy-based silver pastes and screen meshes were investigated to reduce the roughness of the printed layers without interlayer connection defects. Also, the insulating layer showed sufficient strength for the applications. The approach in this study could be extended to use with more complex IoT devices as well as in other electronics applications.

## Experimental section

### Materials

For conductive layers (the top and bottom layers), epoxy-based silver conductive inks were purchased from FP (FP Co. Ltd., Rep. of Korea). Table 1 shows the conductive inks’ basic properties. For final printing, the diluted ink with an additional 12 wt% BCA was used. The final components of the used ink included: 77 wt% silver powder flake type (2um), 8wt% epoxy powder blinder (M_W_ = 7500), 1 wt% hardener, 2 wt% dispersants, and other additives. For the insulating layer, the insulating ink (SRF-300PIY, Seoul Chemical Research Lab., Rep. of Korea) having a viscosity of 80–150 Pas was used. In order to inspire a full R2R process, the die-bonding solder paste (DBA-001, SIPKorea Co., Rep. of Korea) having an adhesion force of 70-80 N and sheet resistance of 3.00 mΩ/□ was used.

**Table 1:** Conductive inks properties (from manufacturer).

Ink	Viscosity ($\eta_{50} )$ (mPas)	Thixotropy ($\eta_{5} /\eta_{50} )$	Adhesion (cross-cutting)
Original ink	39,200	4.44	100/100 (PET)
Original ink + flattening agent	42,690	3.58	
Original ink + 3wt% BCA	25,070	5.32	
Original ink + 12wt% BCA	18,450	3.75	

### Design and mesh preparation

In this study, we converted the design (gerber files) for rigid PCBs into masks for printing. For the insulating layer, the information of via-hole drilling was used as the openings in a covering layer. To prepare for the registration control (i.e. alignment between the overlay printed layers), the designs of the layers were scaled with the factors shown in Table 2. ^27^. For printing, the mesh counts were 400, 200, 325, and 325 for the bottom, insulating, top, and solder resist respectively. The respective mesh thickness and emulsion thickness of those meshes are (39 μm, 15 μm), (80 μm, 15 μm), (58 μm, 15 μm), and (58 μm, 15 μm). In screen printing, the convenient way to increase the thickness of the printed layer is to increase the emulsion thickness of the screen mesh. In this way, the thickness of the printed conductive layer and the insulation of the insulating layer could be controlled.

**Table 2:** The scale factor for passive compensation control of registration.

Direction	1st layer	2nd layer	3rd layer	4th layer
Machine direction (MD)	1.00283041	1.00300585	1.00312281	1.00319298
Cross-direction (CD)	0.99619724	0.99596055	0.99572387	0.99548718

### R2R screen printing process

**Figure 10:**
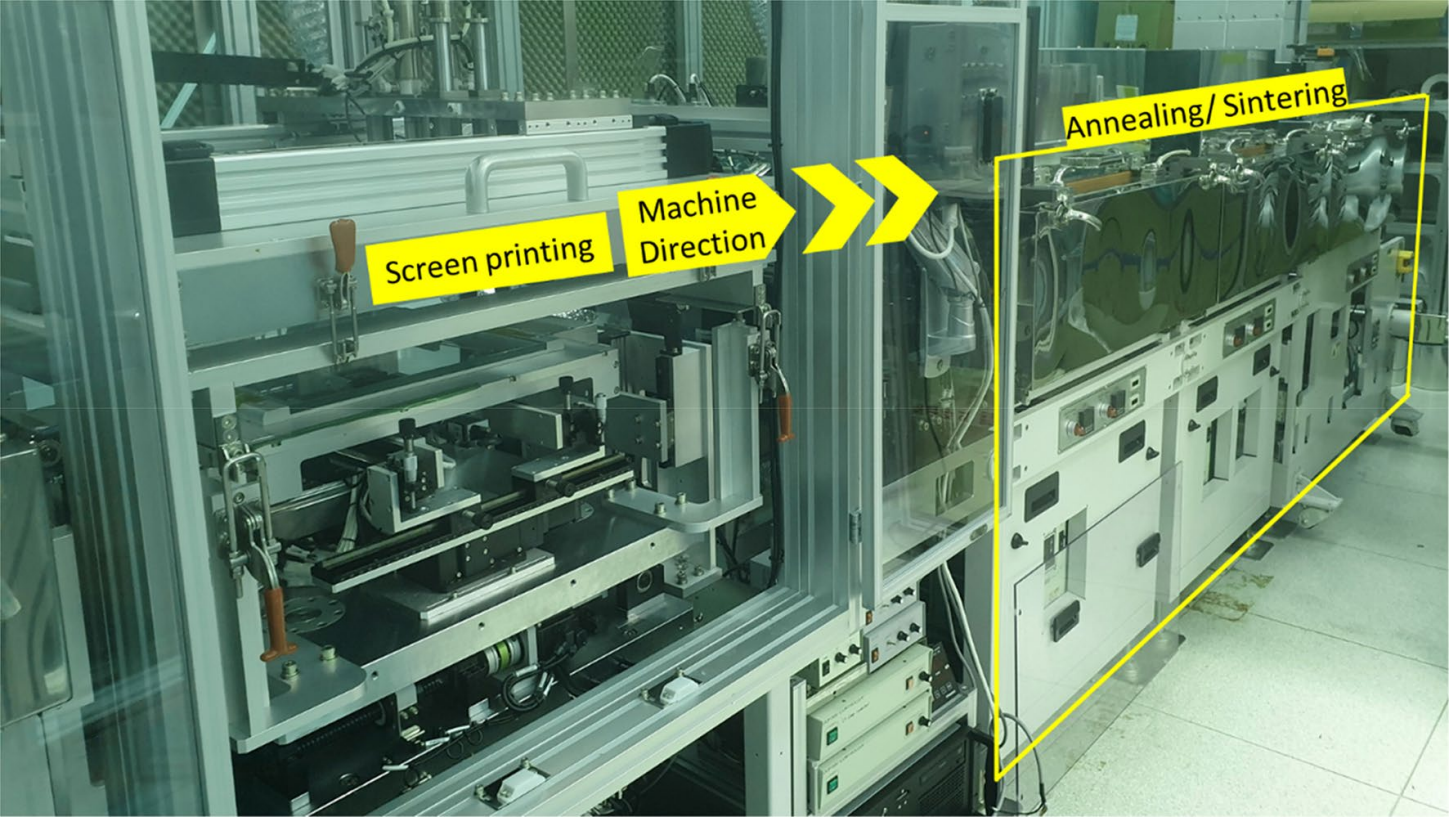
R2R Screen printing system used in the experiments.

An in-house developed R2R screen printing system (Fig. 10) was used for printing. The substrate used in the experiments was PET substrate (web) with a thickness of 125 μm and a width of 70 mm (SKC “Skyrol®” PET SH82, SKC, Rep. of Korea). The registration control (alignment of the overlay printed layers) combined three processes of pre-annealing, passive compensation, and active compensation^27^. Initially, the web substrate was fed continuously for pre-annealing with 130 °C and tension of 3 kgf. The feeding speed was 12 mm/s and the annealing travel was about 3 m. For passive compensation, the factors that influence the substrate deformation by machine direction (the direction along the motion path of the roll) and cross direction (the direction across the printing substrate) were investigated and interpolated to scale the dimensions of the design. During printing, the cameras found the position of the alignment marks, then the tension control system controlled the web tension to actively adjust the printing position for alignment^27,29^. The printing force at the middle of the printed pattern for the bottom, top, and solder resist was 13 kgf, and for the insulating layer was 11 kgf. For all printing, printing speed (squeegee speed) and off-contact gap were 100 mm/s and 3 mm. After printing, the printed layers were sintered in line by sintering chambers with an average temperature of about 130 °C. The time interval between two consecutive printing was used to control the sintering duration. For the final sample, the sintering and annealing times were controlled to be around 10 min by using a time interval between 2 consecutive prints.

### Printing characteristic

The resistivity of the printed patterns was measured through the thickness and sheet resistance as $\rho = R_{s} t$, where $\rho$ is the resistivity, $R_{s}$ is sheet resistance measured by FPP-RS8 (Dasoleng, Rep. of Korea), and *t* is the thickness of the printed pattern measured by Alpha-step (KLA-Tencor D60, KLA Corp., USA). The roughness of the printed pattern was measured by Alpha-step (KLA-Tencor D60, KLA Corp., USA) under ISO-4287 standard. The insulating dielectric property was measure through an LCR meter (Agilent, Keysight Technologies, USA) under ASTM-D150 standard. The IV-curve and breakdown voltage were measured using Agilent 4200 (Keysight Technologies, USA). The linewidth and magnified images of the printed pattern were taken using a confocal optical microscope (Optelics C130, Lasertech, Japan). For cross-section and microstructure investigation, the samples were molded, cross-cut, and observed under an optical microscope and scanning electron microscope (SEM, Hitachi SU8230, Hitachi, Japan).
